# Superconducting- and Graphene-Based Devices

**DOI:** 10.3390/nano12122055

**Published:** 2022-06-15

**Authors:** Filippo Giubileo

**Affiliations:** CNR-SPIN Salerno, 84084 Fisciano, Italy; filippo.giubileo@spin.cnr.it

This Special Issue has been organized to collect new or improved ideas regarding the exploitation of superconducting materials, as well as graphene, aiming to develop innovative devices. For instance, several graphene applications can be enhanced by modifying their surface to introduce a non-zero bandgap, tune adhesion and/or hydrophobicity/hydrophilicity, etc. Similarly, contact resistance in graphene-based field effect transistors can be improved by irradiation [1], leading to an improved device performance. Wei Qin et al. [2] conducted a detailed theoretical investigation using first-principles calculations of “Lithium Diffusion in Silicon Encapsulated with Graphene”. They considered monolayer graphene on silicon substrate to simulate the Si microparticles that were encapsulated in a graphene cage, which can be exploited as anodes in lithium-ion batteries. They demonstrated that defective graphene strongly reduces the energy barriers for Li diffusion in Gr or Gr/Si. Abid et al.’s [3] report, entitled “Interface Kinetics Assisted Barrier Removal in Large Area 2D-WS_2_ Growth to Facilitate Mass Scale Device Production”, employed chemical vapor deposition technique to synthesize mono- and few-layer WS_2_ with areas up to cm^2^ on graphene-oxide-coated Si/SiO_2_ substrates. They show that as-developed WS_2_ layers are polycrystalline (mono- and few-layer), with single-crystal domains that are triangular and hexagonal in shape. Alejandro Toral-Lopez et al.’s [4] report, on “GFET Asymmetric Transfer Response Analysis through Access Region Resistances”, aimed to exploit graphene-based devices to increase the functionality of Si-technology in the field of radio-frequency electronics. They conducted an in-depth investigation of the role of access regions on the performance of graphene-based field effect transistors (GFETs). They demonstrated that the access region conductivity can be tuned by the back-gate bias, improving the RF performance. Graphene represents a prototype of 2D materials and is still widely investigated. Many layered materials, such as the transition metal dichalcogenides, are largely studied for their use as a conducting channel in nanometric field effect transistors, including MoS_2_ [5,6,7], ReSe_2_ [8], WSe_2_ [9], etc. Regarding superconducting-based devices, Jose C. Verde et al.’s [10] report is entitled “Calculations of Some Doping Nanostructurations and Patterns Improving the Functionality of High-Temperature Superconductors for Bolometer Device Applications”. They propose that high-temperature superconductors (HTS) can be nanostructured (and patterned) to obtain an increased functionality as sensing materials for resistive transition-edge bolometer devices (TES). Calculations have been performed to consider the spatial variations in carrier doping into the CuO_2_ planes of the YBaCuO perovskite superconductor, demonstrating an improvement in the bolometric parameters with respect to conventional, nonstructured HTS materials. Paola Romano et al. [11] report, “Transport and Point Contact Measurements on Pr_1−x_Ce_x_Pt_4_Ge_12_ Superconducting Polycrystals” demonstrated that the material has a collective pinning regime with a quasi-2D character for a Ce-doping of about x = 0.07. Moreover, while investigating the properties of metal/superconductor nano-junctions, they showed that the observed conductance features are explained in terms of a superconducting-order parameter with nodal directions, as well as a sign change in the momentum space. Indeed, numerical simulations reported in the framework of Blonder–Tinkham–Klapwijk model show that s-wave pairing and anisotropic s-wave are unsuitable for the reproduction of experimental data obtained at a low temperature. Carlo Barone et al.’s [12] report is entitled “Current-Resistance Effects Inducing Nonlinear Fluctuation Mechanisms in Granular Aluminum Oxide Nanowires”. They measured electric transport and voltage fluctuations in the normal state and in the temperature range of 8–300 K, observing both nonlinear resistivity and two-level tunneling fluctuators. This study helps to improve the fabrication process, therefore reducing the possible sources of decoherence in the superconducting state. This is crucial in quantum technology applications. Sergio Pagano et al.’s [13] report is entitled “Iron-Based Superconducting Nanowires: Electric Transport and Voltage-Noise Properties”. In this work, they fabricated ultra-thin Co-doped BaFe_2_As_2_ nanowires and characterized their transport and intrinsic noise properties. They also investigated the ageing effect on device degradation by means of noise spectroscopy. Interestingly, iron-based superconducting nanowire detectors have several advantages, due to their high operating temperature, when used as innovative single-photon detectors working in the visible and infrared spectral region.
